# Overlap microtubules link sister k-fibres and balance the forces on bi-oriented kinetochores

**DOI:** 10.1038/ncomms10298

**Published:** 2016-01-05

**Authors:** Janko Kajtez, Anastasia Solomatina, Maja Novak, Bruno Polak, Kruno Vukušić, Jonas Rüdiger, Gheorghe Cojoc, Ana Milas, Ivana Šumanovac Šestak, Patrik Risteski, Federica Tavano, Anna H. Klemm, Emanuele Roscioli, Julie Welburn, Daniela Cimini, Matko Glunčić, Nenad Pavin, Iva M. Tolić

**Affiliations:** 1 Max Planck Institute of Molecular Cell Biology and Genetics, Pfotenhauerstrasse 108, 01307 Dresden, Germany; 2 Department of Physics, Faculty of Science, University of Zagreb, Bijenička cesta 32, 10000 Zagreb, Croatia; 3 Division of Molecular Biology, Ruđer Bošković Institute, Bijenička cesta 54, 10000 Zagreb, Croatia; 4 Department of Biological Sciences, Virginia Tech, 1015 Life Science Circle, Blacksburg, Virginia 24061, USA; 5 Virginia Bioinformatics Institute, Virginia Tech, 1015 Life Science Circle, Blacksburg, Virginia 24061, USA; 6 Wellcome Trust Centre for Cell Biology, School of Biological Sciences, University of Edinburgh, Michael Swann Building, Max Born Crescent, Edinburgh, EH9 3BF Scotland, UK

## Abstract

During metaphase, forces on kinetochores are exerted by k-fibres, bundles of microtubules that end at the kinetochore. Interestingly, non-kinetochore microtubules have been observed between sister kinetochores, but their function is unknown. Here we show by laser-cutting of a k-fibre in HeLa and PtK1 cells that a bundle of non-kinetochore microtubules, which we term ‘bridging fibre', bridges sister k-fibres and balances the interkinetochore tension. We found PRC1 and EB3 in the bridging fibre, suggesting that it consists of antiparallel dynamic microtubules. By using a theoretical model that includes a bridging fibre, we show that the forces at the pole and at the kinetochore depend on the bridging fibre thickness. Moreover, our theory and experiments show larger relaxation of the interkinetochore distance for cuts closer to kinetochores. We conclude that the bridging fibre, by linking sister k-fibres, withstands the tension between sister kinetochores and enables the spindle to obtain a curved shape.

## Abstract (web summary)

During metaphase, k-fibre microtubules exert force on kinetochores, but there are also non-kinetochore microtubules close to kinetochores without a known function. Here the authors show that these microtubules, which they call bridging fibres, balance interkinetochore tension by bridging sister k-fibres.

## Graphical abstract

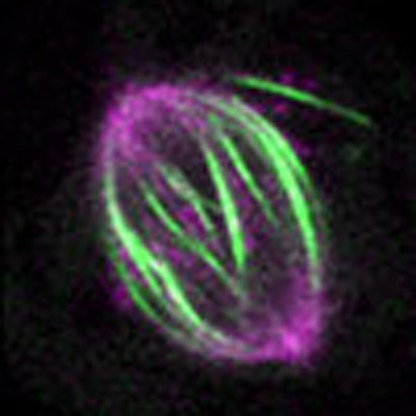

At the onset of division, the cell forms a spindle, a precise self-constructed micromachine based on microtubules (MTs) and MT-associated proteins, which divides the chromosomes between the two nascent daughter cells. The attachment of MTs to chromosomes is mediated by kinetochores, which are protein complexes on the chromosome^1^. MTs generate forces on kinetochores, which are responsible for kinetochore congression to the metaphase plate, silencing of the spindle assembly checkpoint^2^,^3^,^4^ and segregation of sister kinetochores in anaphase.

Spindle MTs can be divided into two major classes with respect to whether they end at the kinetochore (kMTs) or not (non-kMTs). kMTs form parallel bundles known as k-fibres. Likewise, non-kMTs form parallel bundles, but some of them interact with other non-kMTs extending from the opposite spindle pole, thereby forming antiparallel overlap zones, hence they are known as overlap MTs. During metaphase, when kinetochores are bi-oriented, which means that sister kinetochores are attached to k-fibres extending from opposite poles, k-fibres pull on kinetochores^5^. However, non-kMTs have been observed in the vicinity of k-fibres and between sister kinetochores in metaphase^6^,^7^,^8^, which opens an interesting possibility that they may link sister k-fibres and balance the forces on kinetochores. Yet, the function of these non-kMTs is unknown.

Here we show that a bundle of non-kMTs, which we term ‘bridging fibre', bridges sister k-fibres and balances the tension between sister kinetochores. We uncover a strong connection between the bridging fibre and sister k-fibres by cutting a k-fibre with a laser, after which the bridging fibre moves together with the k-fibres and kinetochores. The central part of the bridging fibre contains PRC1 and EB3, suggesting that this bundle consists of antiparallel dynamic MTs. By combining a novel model with experiments, we determine the forces at the kinetochore. Moreover, our theory and experiments show a larger relaxation of the interkinetochore tension for cuts closer to the kinetochore, implying that the tension on kinetochores is generated in a MT length-dependent manner. Thus, we conclude that the bridging fibre, by linking sister k-fibres, withstands the tension between sister kinetochores and supports the rounded shape of the spindle.

## Results

### Bridging MTs are linked with sister k-fibres

**Figure 1:**
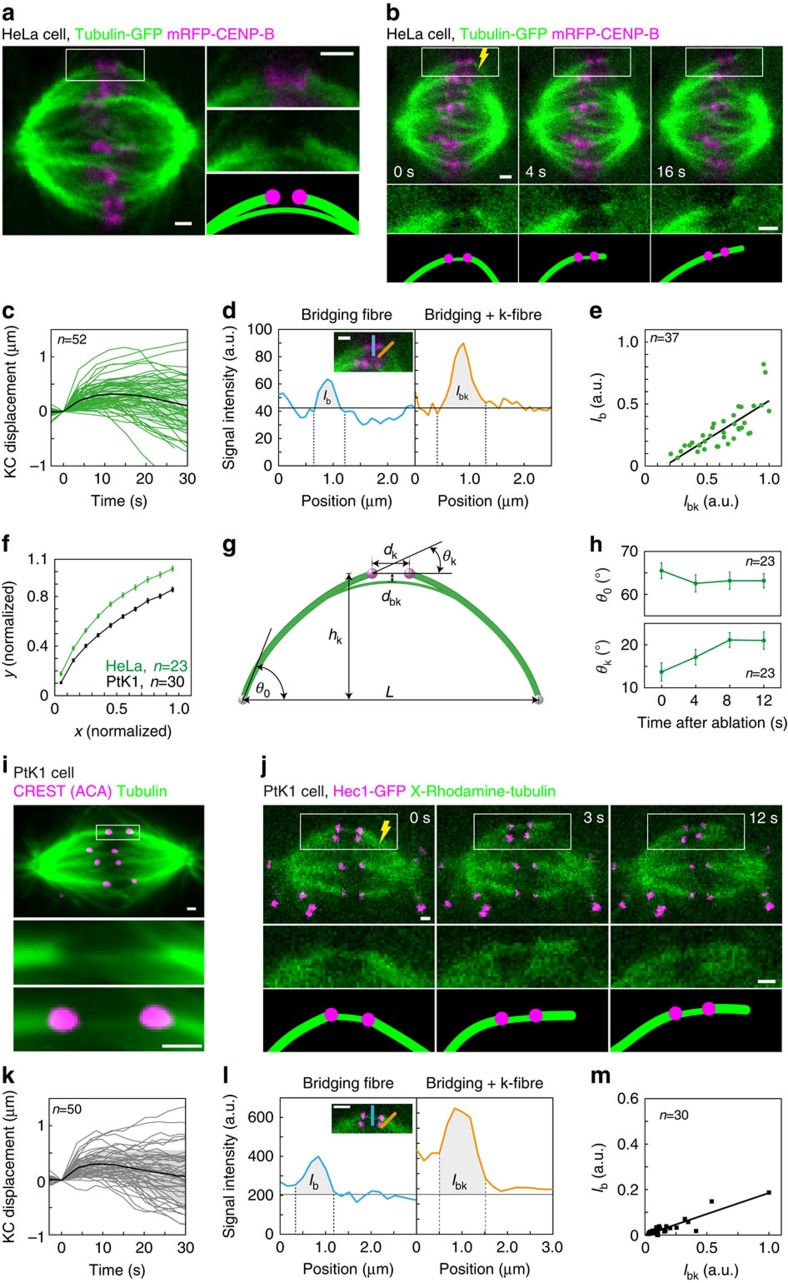
Bridging MTs are linked with sister k-fibres and sister kinetochores. (**a**) Spindle in a HeLa cell expressing tubulin-GFP (green) and mRFP-CENP-B (magenta). Enlargements of the boxed region (top: merge, middle: GFP, bottom: scheme) show a bridging fibre connecting sister k-fibres. (**b**) Time-lapse images of the spindle (top) in a HeLa cell (as in **a**), and enlargements of the boxed region (middle: GFP, bottom: schemes). After the cut (yellow), the bridging fibre moved together with sister kinetochores. (**c**) Displacement of the kinetochore proximal to the cut in the direction perpendicular to the spindle long axis, with respect to its position before the cutting (time 0), in HeLa cells. Individual cells (thin lines), mean (thick line), s.d. (shaded region). (**d**) Tubulin-GFP signal intensity of the bridging fibre, *I*_b_ (blue, measured along the blue line in the image), and the bundle consisting of the bridging and the k-fibre, *I*_bk_ (orange, measured along the orange line), in a HeLa cell. Horizontal lines mark the background signal, vertical lines delimit the area (grey) where the signal was measured. (**e**) *I*_b_ as a function of *I*_bk_ in HeLa cells. (**f**) Normalized shapes (each spindle was scaled so that the pole is at *x*=0 and kinetochore at *x*=1) of a k-fibre in HeLa (green) and PtK1 (black) cells. (**g**) Scheme of the measured parameters of the spindle, see Table 1. (**h**) Angles *θ*_0_ and *θ*_k_ in HeLa cells as a function of time after cutting. (**i**) Spindle in a PtK1 cell (top) immunostained for tubulin (green) and kinetochores (magenta). Enlargements of the boxed region (middle: tubulin, bottom: merge) show a bridging fibre between sister kinetochores. (**j**) Laser-cutting in a PtK1 cell with Hec1-GFP (magenta) and X-Rhodamine-tubulin (green). Legend as in **b**. (**k**) Kinetochore displacement in PtK1 cells. Legend as in **c**. (**l**) Signal intensities *I*_b_ and *I*_bk_ in a PtK1 cell. Legend as in **d**. (**m**) *I*_b_ as a function of *I*_bk_ in PtK1 cells. Note a smaller slope than in **e**, corresponding to fewer MTs in the bridging fibre in PtK1 than in HeLa cells. Scale bars, 1 μm; *n*, number of cells; error bars, s.e.m.

We first set out to determine the organization of MTs along the spindle that interact with a pair of sister kinetochores. We examine only the outermost kinetochores and the associated MTs because they are most easily distinguished from their neighbours in live-cell images. We imaged live metaphase HeLa cells expressing tubulin-GFP (green fluorescent protein) and mRFP-CENP-B (a kinetochore protein). In addition to a strong tubulin-GFP signal along the two k-fibres, we observed a weak signal of non-kMTs appearing as a line connecting these k-fibres, that is, bridging the sister kinetochores (Fig. 1a). We term this subset of non-kMTs ‘bridging MTs' and their bundle a ‘bridging fibre'.

Nevertheless, the close proximity of the bridging fibre to k-fibres and kinetochores does not reveal their interactions. We reasoned that if the bridging fibre is indeed linked with k-fibres and kinetochores, these structures should remain in close proximity and move together in response to the release of force after cutting of a k-fibre. To explore this possibility, we designed a laser-cutting assay to cut k-fibres, similar to previous studies^9^,^10^,^11^,^12^,^13^. In our work, we cut the outermost k-fibre and investigated the resulting movement of the k-fibres and kinetochores (Fig. 1b,c and Supplementary Movie 1). We observed that after cutting, sister kinetochores typically moved outwards from the spindle centre, reaching a maximum displacement of 320±40 nm (results are mean±s.e.m. unless otherwise indicated) in 11 s. Afterwards, the kinetochores started moving back towards the spindle centre (*n*=52 cells, Fig. 1c) in a manner consistent with the results from two recent studies^12^,^13^. Two-dimensional trajectories of the kinetochore that was closer to the cutting site show that this kinetochore moved, on average, along a V-shaped path: first outwards and towards its sister, and subsequently towards the spindle centre (Supplementary Fig. 1a). Importantly, the bridging fibre, the intact k-fibre and the k-fibre stub extending from the kinetochore closer to the cutting site moved together with sister kinetochores (Fig. 1b). The bridging fibre moved together with the k-fibres and kinetochores as a single entity not only during the initial outward-directed movement but also during the movement back towards the spindle (Supplementary Fig. 1b). Similar results were obtained in cells arrested in metaphase using the proteasome inhibitor MG-132 (Supplementary Fig. 1c); thus, we used non-arrested cells in further experiments to minimize cell treatments. On the basis of these results, we conclude that the bridging fibre connects sister k-fibres and kinetochores into a single object.

Next, we estimated the number of MTs in the bridging fibre. We used the signal intensities of the bridging fibre and the k-fibre to determine the relative number of MTs in these two bundles. We measured the signal intensity of the MTs between sister kinetochores, *I*_b_, and across the k-fibre in the vicinity of the kinetochore, *I*_bk_ (Fig. 1d, more examples are shown in Supplementary Fig. 1d). These signal intensities did not vary significantly between consecutive images (Supplementary Fig. 1e). We interpret the signal intensity *I*_b_ as the signal of the bridging fibre, and *I*_bk_ as the sum of the k-fibre signal and the bridging fibre signal, *I*_k_+*I*_b_. We found that, even though the signal intensities of the bridging fibre and of the sum intensity varied from cell to cell, their ratio was roughly constant, *I*_b_/*I*_bk_=45±2% (*n*=37 cells, Fig. 1e and Supplementary Fig. 1f). From this ratio we estimate that the bridging fibre contains *I*_b_/*I*_k_=(*I*_b_/*I*_bk_)/(1−*I*_b_/*I*_bk_)=82±7% of the number of MTs in the k-fibre. A similar value *I*_b_/*I*_bk_=47±3% was measured after laser-cutting, which supports our observation that the bridging MTs remained in close proximity to k-fibres during their movement after the cutting. We measured similar values *I*_b_/*I*_bk_=46±1% (*n*=27 bridging fibres from 20 cells, Supplementary Fig. 1g) in the interior of the spindle (within 3 μm from the long axis of the spindle) on the examples of k-fibres that we could discern from their neighbours. However, we continued our analysis by using only the outermost k-fibres because they are most easily distinguished from other fibres. Previous measurements from electron micrographs have shown that k-fibres in HeLa cells contain *n*_k_=17±2 MTs^14^,^15^. Thus, our data suggest that bridging fibres consist of *n*_b_=14±2 MTs.

To quantitatively describe the shape of the k-fibres, we tracked their contours (Fig. 1f) and extracted the most robust parameters: the angle between the k-fibre and the long axis of the spindle in the vicinity of the spindle pole, *θ*_0_, and in the vicinity of the kinetochore, *θ*_k_, the distance between the bridging fibre and the kinetochore, *d*_bk_, the distance between sister kinetochores, *d*_k_, as well as the spindle length, *L*, and spindle half-width, *h*_k_ (Fig. 1g, Table 1). The bridging fibre was found to be on the inner side of the k-fibres with respect to the spindle centre, 0.24±0.15 μm away from the kinetochores (*n*=42 cells, Supplementary Fig. 1h). We found that the angle at the spindle pole did not change significantly, while the angle at the kinetochore increased by 3.4±1.2 degrees 4 s after the cut (*n*=23 cells, Fig. 1h). The first finding suggests that the k-fibre is clamped in the vicinity of the spindle pole. The two findings together show that the k-fibre became straighter after the cutting, which is consistent with the observed aligning of the k-fibre stub with the intact k-fibre (Fig. 1b). The distance between sister kinetochores decreased after cutting by 0.15±0.06 μm (*n*=52 cells), which is similar to previous studies^12^,^13^, and it indicates that sister kinetochores were under tension and thus bi-oriented before the cut.

To test whether bridging MTs are found also in other mammalian cells, we analysed PtK1 cells. We observed bridging MTs extending between bi-oriented sister kinetochores, both in immunostained PtK1 cells (Fig. 1i) and in live cells expressing Hec1-GFP (a kinetochore protein), which were injected with X-Rhodamine-tubulin (Fig. 1j). After cutting of a k-fibre, the bridging fibre moved together with the intact k-fibre, the k-fibre stub and the sister kinetochores in a direction away from the spindle centre (Fig. 1j,k and Supplementary Movie 2). The ratio of the tubulin signal intensity in the bridging fibre to the sum signal of the k-fibre and the bridging fibre was *I*_b_/*I*_bk_=20±2% (*n*=30 cells, Fig. 1l,m and Supplementary Fig. 1i). As k-fibres in PtK1 cells in metaphase consist of *n*_k_=24±5 MTs^6^,^16^, our data imply that bridging fibres in PtK1 cells consist of *n*_b_=6±1 MTs. This value is in agreement with a previous estimate that, on average, three to eight MTs are laterally associated with a kinetochore in PtK1 cells, based on electron tomography data^17^. Taken together, our results show that bridging MTs are present and display similar behaviour in different mammalian spindles.

### PRC1 and EB3 are found in the bridging fibres

**Figure 2:**
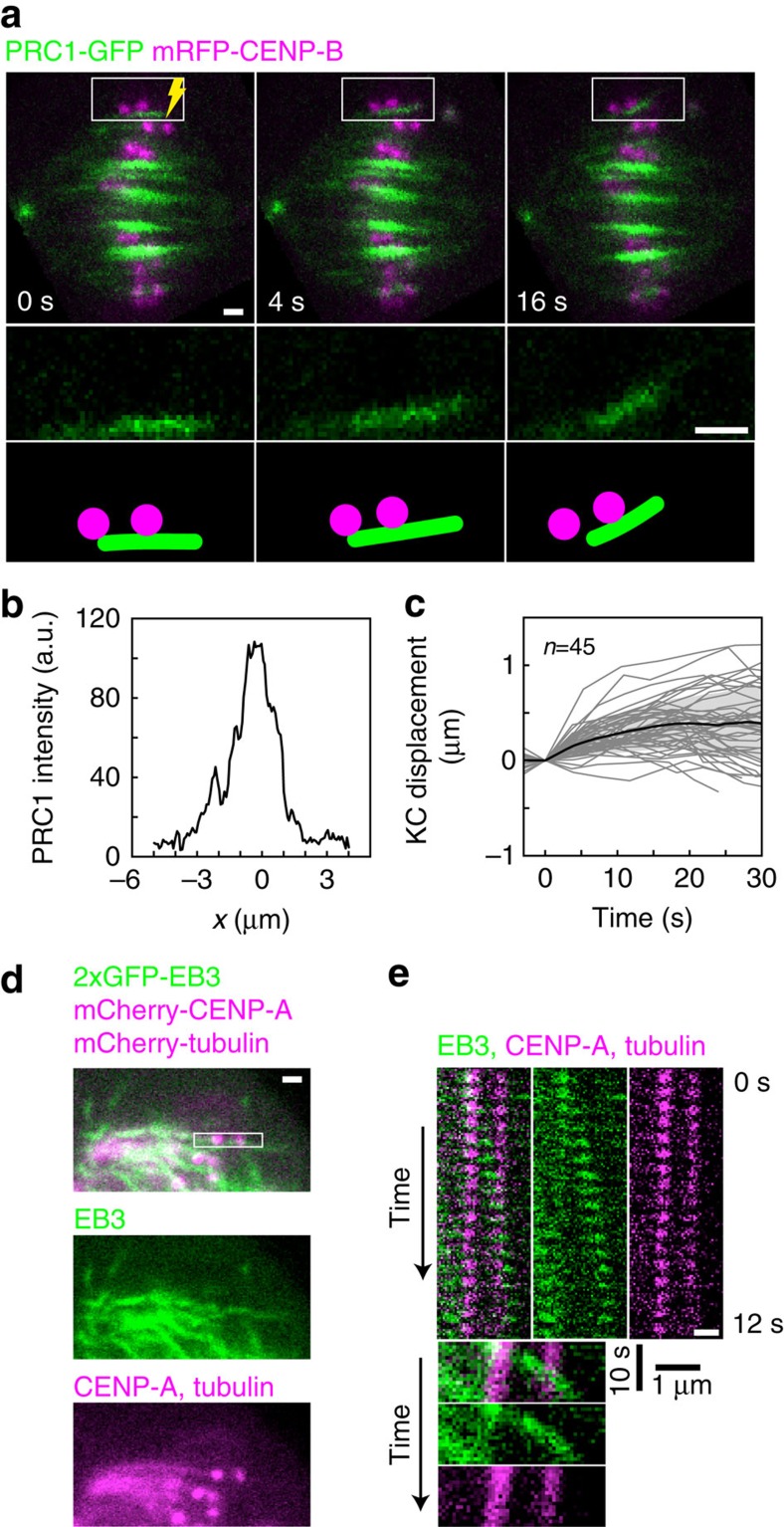
PRC1 and EB3 are found in the bridging fibres. (**a**) Time-lapse images of the spindle (upper row) in a HeLa cell expressing PRC1-GFP (green) and mRFP-CENP-B (magenta), enlargements of the region inside the white rectangle (PRC1-GFP only, middle) and the corresponding drawings (bottom). The outermost k-fibre was cut with a laser (yellow lightning sign) at time 0. After the cut, the PRC1 signal moved together with sister kinetochores. (**b**) Measurement of the PRC1-GFP signal intensity along a curved line drawn by following the outermost spindle MTs in a HeLa cell. 0 on the *x* axis marks the midpoint between sister kinetochores. (**c**) Displacement of the kinetochore that was closer to the cut site in the direction perpendicular to the long axis of the spindle, with respect to its position before the cutting, in HeLa cells expressing PRC1-GFP and mRFP-CENP-B. Individual cells, the mean value and the s.d. are shown by thin lines, thick black line and the shaded region, respectively; *n* represents the number of cells. (**d**) Time projection over 7 s of images of a U2OS cell expressing 2 × GFP-EB3, mCherry-CENP-A and mCherry-tubulin. Merged image after the cutting, which was performed on the right side of the boxed kinetochore pair, and separate channels are shown. Traces of numerous EB3 comets are visible in the middle panel. The location of the intact k-fibre attached to the boxed kinetochore is visible in the bottom panel and was used to identify the comets travelling along this k-fibre and the associated bridging fibre. (**e**) Time-lapse images showing the region inside the white rectangle in **d** (top) and maximum-intensity projections of these images on the *x* axis (bottom). The images were taken at 0.77-s intervals. Merged images and separate channels are shown. An EB3 comet is visible as a green spot moving to the right in the time-lapse images, and as the oblique green line in the maximum-intensity projections. The comet is passing between the sister kinetochores (magenta). White scale bars, 1 μm.

To test whether bridging fibres consist of antiparallel MTs, we explored whether PRC1, a highly conserved MT-associated protein that binds to overlap zones of antiparallel MTs^18^, is found in the bridging fibre. We used our laser-cutting assay on HeLa cells stably expressing PRC1-GFP from a BAC^19^ and mRFP-CENP-B (Fig. 2a). We observed PRC1 signal along the spindle with enrichment in its central part (Fig. 2a; see Supplementary Fig. 2a for immunostaining of endogenous PRC1), and that this signal increased at the transition from metaphase to anaphase (Supplementary Fig. 2b), as described in previous studies^20^,^21^,^22^. Intensity profiles of the PRC1-GFP signal along a curved line following the outermost MTs of the metaphase spindle show that the overlap zones of antiparallel MTs extend over a region of 4.5±0.2 μm in the vicinity of sister kinetochores (*n*=22 cells, Fig. 2b). After laser-cutting of a k-fibre, sister kinetochores moved together with the PRC1 signal away from the spindle centre (*n*=45 cells, Fig. 2a,c and Supplementary Movie 3). This result shows that the PRC1 signal closest to the kinetochores corresponds to the central part of the bridging fibre where antiparallel MTs overlap. In a similar approach, we used cells expressing GFP-tagged KIF11, a human kinesin-5, also known as Eg5, a motor protein that crosslinks antiparallel MTs in the spindle and slides them apart by walking towards their plus ends^23^,^24^,^25^. We found that after cutting, the signal of KIF11 on the bridging fibre moved together with k-fibres and kinetochores (*n*=30 cells, Supplementary Fig. 2c). Collectively, these results further support the conclusion that the bridging fibre connects sister k-fibres, and indicate that this fibre consists of antiparallel MTs.

Next, we wanted to test whether bridging fibres contain growing MTs. To observe the plus ends of growing MTs, we used a U2OS cell line stably expressing 2xGFP-EB3, a plus end marker^26^, mCherry-CENP-A to mark the kinetochores and mCherry-tubulin (Fig. 2d). To ensure that we are following the plus ends of MTs interacting with a single pair of sister kinetochores, we used our laser-cutting assay to displace the outermost sister k-fibres and the associated bridging fibre away from the neighbouring k-fibres (Supplementary Movie 4). Time projections of the acquired movies allowed us to identify the location of the cut k-fibre, the intact sister k-fibre and the corresponding bridging fibre, and thus to distinguish these fibres from other MT populations such as astral MTs (Fig. 2d). EB3 comets that moved along the k-fibres and crossed the region between the sister kinetochores were identified as those belonging to the bridging fibre, whereas the comets moving along the k-fibres but stopping at the first sister kinetochore were identified as those within the k-fibre. We measured 1.9±0.4 comets per minute moving within the bridging fibre in the direction towards the cut site (Fig. 2e) and 0.6±0.2 comets per minute moving in the opposite direction (*n*=26 and 8 comets, respectively, in 16 cells). This result provides independent evidence for the existence of bridging MTs and shows that the bridging fibre contains dynamic antiparallel MTs. We also measured 2.0±0.4 comets per minute moving within the intact k-fibre towards the kinetochore, and 0.8±0.2 comets per minute moving within the k-fibre stub towards the kinetochore (*n*=28 and 11 comets, respectively). These data indicate that bridging and k-fibres have a comparable number of growing MTs. The EB3 comets in the bridging fibre moved with a velocity of 13.1±1.1 μm min^−1^ (*n*=16), while the comets in the k-fibre had a velocity of 13.3±1.2 μm min^−1^ (*n*=12). In the same cells, the comets on astral MTs moved at 11.9±0.3 μm min^−1^ (*n*=70), consistent with previous measurements^27^. The similar velocities of the EB3 comets on bridging, kinetochore and astral MTs suggest that the polymerization of these three groups of MTs is regulated in a similar manner.

### Theoretical model

**Figure 3:**
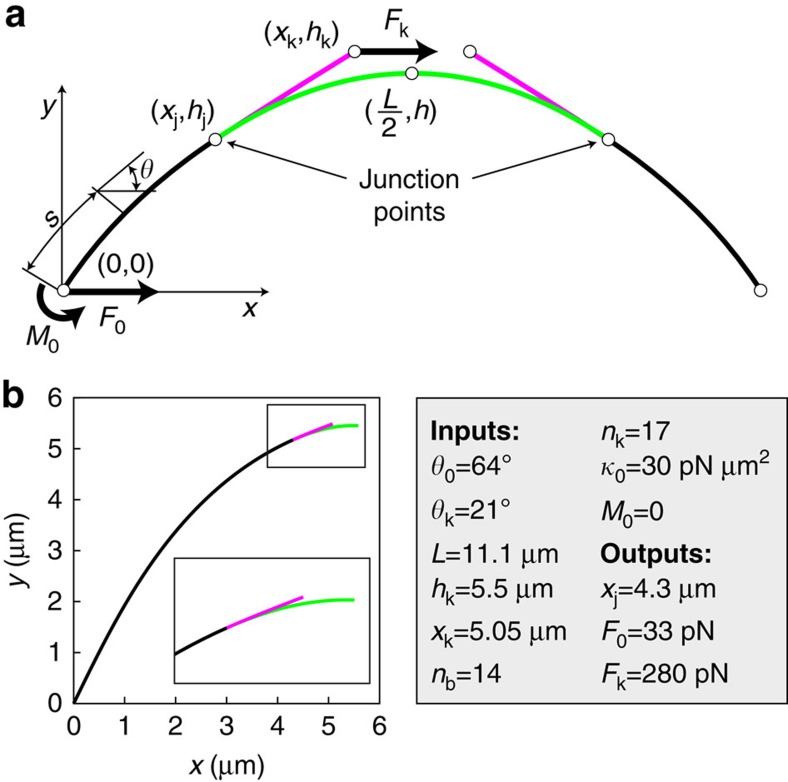
Theoretical model. (**a**) Scheme of the model. MT bundles are represented by three slender rods described by tangential angle, *θ*(*s*), along the contour length, *s*. The first one (black) extends between coordinates (0,0) and (*x*_j_,*h*_j_), which represent positions of the spindle pole and the junction, respectively. The second rod (magenta) extends between the junction and the coordinate that represents the position of the kinetochore, (*x*_k_,*h*_k_). The third rod (green) extends between the junction and the coordinate that represents the midpoint of the system (*L*/2,*h*). The forces at the spindle pole and at the kinetochore are denoted *F*_0_ and *F*_k_, respectively, and the bending moment at the spindle pole is denoted by *M*_0_. (**b**) Predicted shape of the bridging fibre and the k-fibres for the parameters given next to the graph. Note that *M*_0_ is set to 0 because it has a complementary contribution to *F*_0_ (Supplementary Fig. 3a). The region inside the box is enlarged in the inset; colour code as in **a**.

To examine the role of the bridging fibre in the force balance on kinetochores, we introduce a theoretical model that includes the bridging fibre as a link between sister k-fibres (Fig. 3a, Methods). The model takes into account the elastic properties of MT bundles and the forces acting at their ends. In general, forces may induce buckling of an elastic rod; thus, the shape of the k-fibres and the bridging fibre depends on the magnitude of the forces at their ends. A similar approach was used previously to calculate the shape of overlap MT bundles^28^. A mathematical formulation of our model is given in Methods. Briefly, our model describes two k-fibres and the bridging fibre as elastic rods, which merge at two junction points (Fig. 3a). The tension between sister kinetochores is described as a pulling force acting at the end of the k-fibre that corresponds to the position of the kinetochore. In the model, we describe our experimental finding that the end points of the k-fibres are clamped at the location of the spindle poles by introducing bending moments acting at the poles.

Even though the shape of the k-fibres and the bridging fibre is a consequence of the forces acting at their ends, we used the shape of these fibres to calculate the forces. The reason for this reverse approach is that in our experiments we measured the shape of the k-fibres and the bridging fibre, whereas we were not able to measure the forces. Our precise measurements of the shape allowed us to calculate also the position of the junction, which is a novel ingredient introduced in our model, despite being experimentally inaccessible in live-cell imaging. In particular, we used as input parameters for the model the angle at the spindle pole, the angle at the kinetochore, the spindle length and width, the horizontal position of the kinetochore and the number of MTs in the bridging fibre measured here (Fig. 1g and Table 1), as well as the known number of MTs in the k-fibre^14^,^15^ and the known flexural rigidity of a single MT^29^. Thus, by using the measured geometry of the spindle and the known elasticity of MTs, we obtain as the output of the model the position of the junction and the forces at the spindle pole and at the kinetochore.

We solve the model equations as described in Methods. For the set of parameters given in Fig. 3b, the model predicts that the junction is positioned 0.75 μm away from the kinetochore. Note that the kinetochores are above the bridging fibre (Fig. 3b), in accordance with the experimental measurements (Table 1). The model predicts that the force at the spindle pole, *F*_0_=33 pN, acts inwards, resulting in the compression and buckling of the bridging fibre. The predicted tension at the kinetochore is *F*_k_=280 pN, which propagates along the segment of the k-fibre between the junction and the kinetochore. The two inward forces are counteracted by the compression in the central segment of the bridging fibre between the two junctions. The results of the model are robust on the variation of the input geometric parameters within the experimental variability (Supplementary Fig. 3). We conclude that the central part of the bridge balances forces acting at the pole and at the kinetochore, allowing coexistence of tension and compression within a single k-fibre.

### Force at the pole increases with the bridging fibre thickness

**Figure 4:**
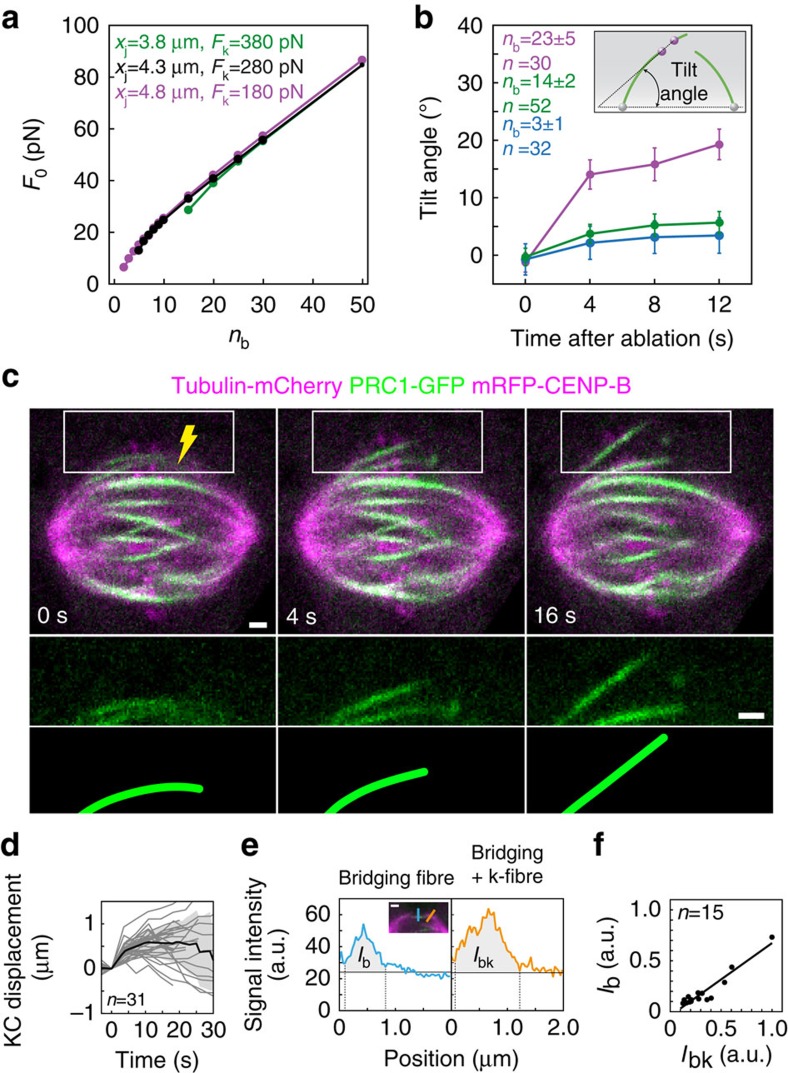
Force at the spindle pole increases with the number of MTs in the bridging fibre. (**a**) Force at the pole, *F*_0_, as a function of the number of MTs in the central part of the bridging fibre, *n*_b_. The inputs of the model are *θ*_0_=64°, *L*=11.1 μm, *x*_k_=5.05 μm, *n*_k_=17, *κ*_0_=30 pN μm^2^, *M*_0_=0, *x*_j_=4.3 μm and *F*_k_=280 pN (black). Changing the values of the least known parameters has a minor influence: *x*_j_=4.8 μm and *F*_k_=180 pN (pink), *x*_j_=3.8 μm and *F*_k_=380 pN (green). Note that, in addition to *F*_0_, the outputs of the model are *θ*_k_ and *h*_k_ (not shown). (**b**) Tilt angle of the sister kinetochores with respect to the spindle axis (see scheme), as a function of time after cutting. HeLa cell lines with different number of MTs in the bridging fibre, *n*_b_ (see legend): cells expressing PRC1-GFP, mRFP-CENP-B and tubulin-mCherry (pink), see Fig. 4c–f; cells expressing GFP-tubulin and mRFP-CENP-B (green), see Fig. 1a–e; and cells expressing GFP-tubulin and mRFP-CENP-B, treated with PRC1 siRNA (blue), see Supplementary Fig. 4. (**c**) Time-lapse images of the spindle (top) after the cut (yellow) in a HeLa cell expressing PRC1-GFP (green), mRFP-CENP-B and tubulin-mCherry (both in magenta), enlargements of the boxed region in the GFP channel (middle) and the corresponding drawings (bottom). (**d**) Displacement of the kinetochore that was closer to the cut in the direction perpendicular to the spindle long axis, with respect to the position before the cutting (time 0), in HeLa cells as in **c**. Individual cells (thin lines), mean value (thick line) and s.d. (shaded region). (**e**) Tubulin-mCherry signal intensity of the bridging fibre, *I*_b_ (blue, measured along the blue line in the image), and of the bundle consisting of the bridging and the k-fibre, *I*_bk_ (orange, measured along the orange line), in a fixed HeLa cell expressing PRC1-GFP and tubulin-mCherry. Horizontal lines mark the background signal and vertical lines delimit the area (grey) where the signal was measured. (**f**) *I*_b_ as a function of *I*_bk_ in HeLa cells as in **c**. Scale bars, 1 μm; *n*, number of cells; error bars, s.e.m.

We used the model to investigate how important is the bridging fibre for the force balance in the k-fibres. To this end, we solved the model for different numbers of MTs in the bridging fibre and found that a bridging fibre with more MTs results in larger force at the spindle pole, and consequently a larger force in the bridging fibre (Fig. 4a). This result is intuitive because a larger force is needed to bend an elastic element with a higher flexural rigidity.

To experimentally test the model prediction that the force at the pole increases with the number of MTs in the bridging fibre, we compared the response to laser-cutting in cells with different numbers of MTs in the bridging fibre. We reasoned that the force at the pole, which is released by laser-cutting, drives the movement of the bridging fibre and the k-fibres together with the attached chromosomes through the viscous cytoplasm, as the fibres straighten. Thus, if the released force is larger, the straightening will be faster. To experimentally quantify how fast the bridging fibre and the k-fibres straighten after the cut, we measured how much sister kinetochores are tilted with respect to the spindle axis (see scheme in Fig. 4b). Sister kinetochores are roughly parallel with the spindle axis in the intact spindle, whereas they become tilted after cutting. We chose this measure of straightening because the positions of kinetochores were determined with higher precision than the positions of MTs in our experiments.

We found that in our original cell line, which has *n*_b_=14±2 MTs in the bridging fibre (Fig. 1b,d,e), sister kinetochores became tilted by 4.0±0.7 degrees 4 s after the cut, that is, the bridging fibre and the k-fibres became straighter (Fig. 4b). We next studied the response to laser-cutting in cells with thicker bridging fibres, by using a cell line expressing mCherry-tubulin, PRC1-GFP and mRFP-CENP-B (Fig. 4c,d and Supplementary Movie 5). In this cell line, which had *n*_b_=23±5 MTs in the bridging fibre (Fig. 4e,f), sister kinetochores became tilted by 15.9±2.1 degrees 4 s after the cut (Fig. 4b). Thus, the bridging fibre and the k-fibres straightened faster in cells with thicker bridging fibres. On the contrary, in a cell line with thinner bridging fibres containing *n*_b_=3±1 MTs, which was obtained by PRC1 short interfering RNA (siRNA; see Methods and Supplementary Fig. 4a), sister kinetochores became tilted by 2.9±1.0 degrees after the cut (Fig. 4b), indicating a slightly slower straightening of the fibres in comparison with our original cell line. Consistent with these results, we observed that the k-fibre stub became more aligned with the intact k-fibre in cells with thicker bridging fibres (Supplementary Fig. 4b). We conclude that the force at the spindle pole and consequently in the bridging fibre increases with the fibre thickness.

### Bridging MTs contribute to the interkinetochore tension

**Figure 5:**
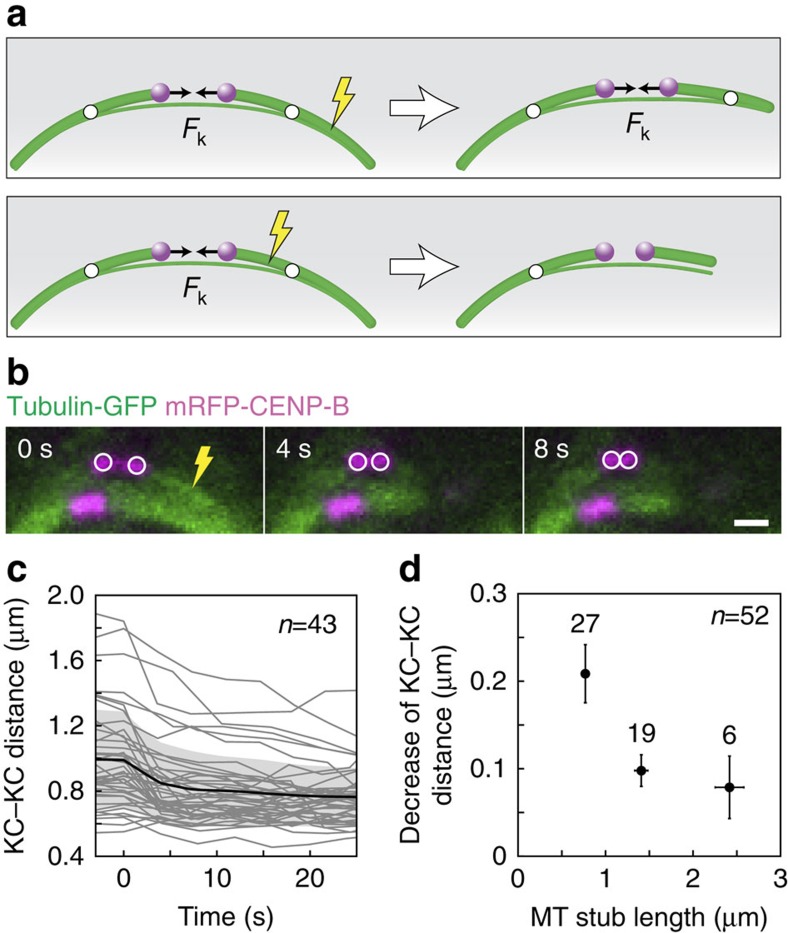
Bridging MTs contribute to the generation of tension between sister kinetochores. (**a**) Two different outcomes of the cut depending on the location of the cut (lightning sign) with respect to the junction (white circle), predicted by the model. (**b**) Time-lapse images of a HeLa cell expressing GFP-tubulin (green) and mRFP-CENP-B (magenta) before the cut (top) and after the cut (middle and bottom) showing a decrease in the interkinetochore distance after the cut. Scale bar, 1 μm. (**c**) Interkinetochore distance as a function of time with respect to the time of ablation (time 0) in HeLa cells expressing GFP-tubulin and mRFP-CENP-B. Data from individual cells, the mean value and the s.d. are shown by thin lines, the thick black line and the shaded region, respectively. (**d**) Absolute value of the decrease in the interkinetochore distance 4 s after the cut in the HeLa cell line from **b**,**c** as a function of the length of the MT stub remaining attached to the kinetochore after the cut. The number of cells in each bin is given near the corresponding data point, *n* denotes the total number of cells, error bars represent s.e.m.

We next explored what the model predicts for the tension between sister kinetochores in cases that mimic laser-cutting experiments. There are two distinct cases depending on whether the length of the k-fibre stub is shorter or longer than the distance between the kinetochore and the junction. If the k-fibre stub is longer than this distance, the connection between the stub and the bridging fibre is preserved, and the compression in the bridging fibre between the two junction points keeps balancing the tension between sister kinetochores (Fig. 5a, top). On the contrary, if the k-fibre stub is shorter than the distance between the kinetochore and the junction, the connection between the stub and the bridging fibre is lost; thus, the tension between sister kinetochores drives their movement towards each other until the tension vanishes (Fig. 5a, bottom). Therefore, the theory predicts two different outcomes for the cutting on either side of the junction.

To experimentally explore the dependence of the tension on the location of the cut, we used our laser-cutting experiments. In addition to the information on the force in the bridging fibre, these experiments provide independent information on the change in the distance between sister kinetochores after the cut, which we use as a readout of the change in the tension between them. Sister kinetochores got closer to each other after the cut (Fig. 5b,c), as observed in previous studies^12^,^13^. Importantly, we found that the reduction of the interkinetochore distance was larger for cuts closer to the kinetochore (Fig. 5d), suggesting that the reduction in tension followed the same trend. The reduction in the interkinetochore distance was measured 4 s after the cutting. On the other hand, the transport of the stub towards the pole to which it was originally connected, which is accompanied by an increase in the interkinetochore distance, starts after an average delay of 15 s (refs 12, 13; see also Fig. 1c and Supplementary Fig. 1a). Thus, the trend observed in Fig. 5d cannot be explained by stronger pulling forces acting on longer stubs during their transport towards the pole. We found a transition in the reduction of the interkinetochore distance when the cut was made more than 1 μm away from the kinetochore. This finding is consistent with the prediction from our theory that tension either remains or vanishes depending on the location of the cut with respect to the junction. Importantly, this experiment provides an estimate that the junction is found at a distance of roughly 1 μm away from the kinetochore. This estimate of the location of the junction is independent from the result of the model that the junction is close to the kinetochore (Fig. 3b), which was obtained based on the shape of an intact k-fibre. The two independent tests described in Figs 4 and 5 provide support for our model, which includes a bridging fibre as a link between sister k-fibres, and show that the bridging fibre balances the force on sister k-fibres.

## Discussion

We have shown that a bundle of overlap MTs interacts laterally with sister k-fibres and acts as a bridge between sister kinetochores, for which reason we named this bundle bridging fibre. Interestingly, non-kinetochore MTs that extend in a region between sister kinetochores have been observed previously in electron micrographs of plant endosperm^8^ and Xenopus extracts^7^, and intermingling of non-kinetochore MTs with k-fibres has been seen close to the kinetochore of PtK1 and Chinese hamster ovary cells^6^,^30^,^31^. Nevertheless, the observed close proximity of these MTs does not provide insight into possible interactions. By using laser-cutting experiments we have shown that bridging fibres move together with k-fibres, which suggests that these fibres are linked into a mechanical object able to survive substantial physical perturbations such as severing of k-fibres.

Spindles come in different shapes, depending on the cell type. On the basis of our model, we propose that spindles with a thicker bridging fibre have a curved shape, such as HeLa spindles, whereas those with a thinner bridge have a diamond-like shape, such as PtK1 spindles. Thus, we expect that, as long as all other geometric parameters are comparable, round spindles will display thicker bridging fibres than diamond-like spindles.

Similar to the interactions between bridging and k-fibres found here, previous studies have shown the existence of lateral connections between different MT bundles in the spindle. For example, the observation that neighbouring kinetochore pairs oscillate in a coordinated manner has been explained by elastic linkages that physically link pairs of k-fibres^32^. Similarly, k-fibres have been proposed to be coupled by viscoelastic elements to overlap MTs^33^. Our experiments have shown that after the cutting bridging fibres move together with k-fibres, while the outermost k-fibre separates from its neighbours. Therefore, the links between bridging and k-fibres are stronger and can survive a more severe perturbation than the links between the neighbouring k-fibres.

Forces in the spindle are crucial for the incorporation of chromosomes into the spindle and the segregation of sister chromatids. Even though we did not directly measure these forces, we used the shape of the spindle and the elastic properties of MTs to estimate the forces at the kinetochore and at the spindle pole by using our theoretical model. We found that the tension forces at the kinetochores in HeLa cells are roughly 100–300 pN. This value is smaller than the maximum force of 700 pN measured on a chromosome in anaphase^34^, but larger than 50 pN, which was estimated from measurements of chromosome elongation in prometaphase^35^. In our model, tensile forces at the kinetochore are balanced by the compression in the bridging fibre. Tension between sister kinetochores is required for the passage through the spindle assembly checkpoint^2^,^3^,^36^; thus, it will be interesting to identify the role of bridging MTs in this process.

**Figure 6:**
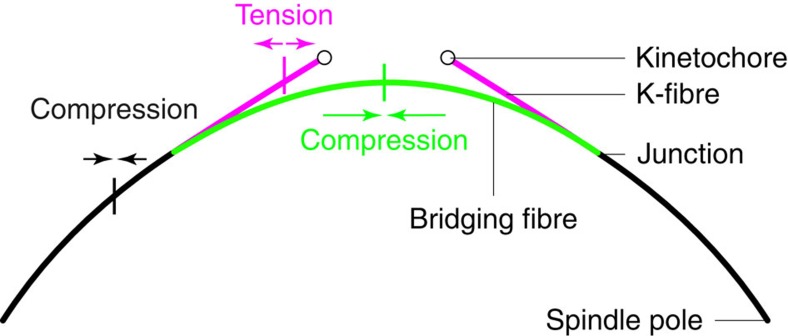
Compression in the bridging fibre balances the tension on the kinetochore. Our theory together with experiments predicts the force balance in the k-fibre and the bridging fibre. The segment of the k-fibre between the junction and the kinetochore (magenta) is under tension, while the bundle consisting of the segment of the k-fibre and the bridging fibre between the pole and the junction (black), as well as the central part of the bridging fibre between the two junctions (green), are under compression. The central part of the bridging fibre balances the forces acting at the pole and at the kinetochore, allowing coexistence of tension and compression within a single k-fibre.

Intriguingly, we found that tension and compression coexist in a single k-fibre. In particular, the segment of the k-fibre between the junction and the kinetochore is under tension, while the bundle consisting of the segment of the k-fibre and the bridging fibre between the pole and the junction, as well as the central part of the bridge between the two junctions, are under compression (Fig. 6). Similarly, it has been proposed that k-fibres are tensed near kinetochores and compressed near poles^37^; however, the underlying mechanism for how the force changes direction along the k-fibre was unclear. Our model, which includes the bridging fibre as a link between sister k-fibres, provides an explanation for this counterintuitive force map because the central part of the bridge balances the forces acting at the pole and at the kinetochore, allowing coexistence of tension and compression within a single k-fibre.

We have observed that PRC1 localizes to the central part of the bridging fibre, which is indicative of overlap regions of antiparallel MTs. In these regions, plus end-directed motors such as kinesin-5 may generate the compressive force in the bridging fibre. PRC1 localization in the bridge, together with the observation of EB3 comets passing between sister kinetochores, suggests that the bridging fibre contains dynamic MTs that link sister k-fibres. Even though the bridging fibre may consist of a few long MTs or many short ones, our conclusions depend on the flexural rigidity of the bridging fibre as a single object, but not on its detailed structure. Future work will reveal the locations of MT plus and minus ends in the bridging fibre.

On the basis of our findings, we speculate about the morphology of the junction. Our model describes the junction in a simplistic manner as a mathematical point in which the k-fibre and the bridging fibre merge while keeping a smooth shape along their entire length. This picture is consistent with our observation that some EB3 comets in the k-fibre travel to the kinetochore, whereas others ‘peel off' the k-fibre and follow the bridging fibre. However, our model does not include MT crosslinkers and their forces, which are most likely responsible for the morphology of the junction. Because the kinetochore is under tension, which pulls the k-fibre inwards, there must be a force on the k-fibre balancing this pulling (Supplementary Fig. 4c). Such force may be generated by the crosslinkers under tension, which link the k-fibre and the bridging fibre. Force balance on the k-fibre is established if the bridging fibre is on the outside of the k-fibre in the region where the fibres are linked by crosslinkers (Supplementary Fig. 4c). On the contrary, the bridging fibre is on the inside of the k-fibre in the central region of the bridge where these fibres are separate, hence the bridging fibre crosses the k-fibre (Supplementary Fig. 4c). We expect the crosslinkers to accumulate all along the interface between the bridging fibre and the k-fibre, rather than predominantly at the junction. Note that during metaphase kinetochores move and k-fibres grow and shrink; thus, the junction may also move. For example, kinetochores may plough through the junction during their oscillations around the metaphase plate^38^. Yet, these speculations await further research.

In summary, we have shown that the bridging fibre balances the forces on kinetochores in metaphase. In addition to this function, the bridging fibre may play a role in the initial interactions between the kinetochores and the spindle MTs, as well as in the separation of sister kinetochores in anaphase, by generating forces via motor-driven sliding of MTs or by acting as mechanical support for the k-fibres. It will be interesting to see, both theoretically and experimentally, to which extent the bridging fibre contributes to different steps of mitosis.

## Methods

### Theory

In our model, one half of the system consists of the bridging fibre and sister k-fibres, while the other half is obtained by imposing reflection symmetry (Fig. 3a). MT bundles are represented as three slender rods described by tangential angle, *θ*(*s*), along the contour length, *s*. The first one, denoted as p, extends between coordinates (0,0) and (*x*_j_,*h*_**j**_), which represent positions of the spindle pole and the junction, respectively. The second rod, denoted as k, extends between the junction and the coordinate that represents the position of the kinetochore, (*x*_k_,*h*_k_). The third rod, denoted as b, extends between the junction and the coordinate that represents the midpoint of the system (*L*/2,*h*). The shape of elastic rods with flexural rigidity, *κ*_i_, obeys Euler–Bernoulli beam equations


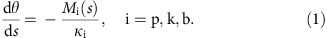


Bending moments are given by *M*_p_=*M*_0_+*F*_0_*y*(*s*), *M*_k_=−*F*_k_(*y*(*s*)−*h*_k_) and *M*_b_=(*F*_k_+*F*_0_)*y*(*s*)−*F*_k_*h*_k_+*M*_0_. Here *F*_0_ and *F*_k_ denote the forces at the spindle pole and at the kinetochore, respectively. Because MTs are clamped at the spindle pole, we also introduce a non-vanishing bending moment at the spindle pole, *M*_0_. The distance from the horizontal spindle axis is denoted by *y*, and 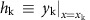.

### Flexural rigidities of the rods

Flexural rigidities of the bridging fibre and the k-fibre have not been measured experimentally; however, the numbers of MTs in these fibres are known. We calculate the flexural rigidities as *κ*_i_=*n*_i_*κ*_0_, where *n*_i_ is the number of MTs in the i-th rod, *κ*_0_ is the flexural rigidity of a single MT and the number of MTs in the rod p is calculated as *n*_p_=*n*_k_+*n*_b_. Here we use the assumption that the MTs in a fibre are allowed to slide with respect to each other as the fibre bends. We have chosen this assumption because the MTs in a k-fibre are connected by a meshwork of linked multipolar connectors that are found at different angles^39^; thus, we expect the meshwork to be remodelled as the MTs slide. However, if MTs are crosslinked in a manner that does not allow for sliding, then the flexural rigidities would scale as the MT number squared^40^.

### Parameterization of the model

In the Cartesian coordinates, (*x*(*s*),*y*(*s*)), for bending moments given above, equation (1) transforms to the set of three second-order nonlinear differential equations:


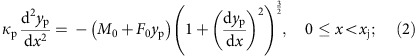



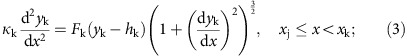



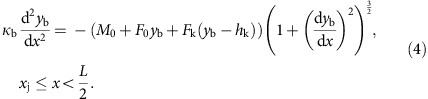


The end points of intervals represent the end points of three rods along the *x* axis. These equations have a unique solution for six initial conditions: two initial conditions at the spindle pole,


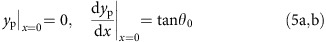


and four initial conditions at the junction, which describe smooth and continuous shape, that is, that the three rods are clamped and tangential to each other at the junction,


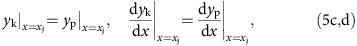



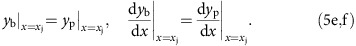


In conclusion, set of equations (2)–(4), 4, 5 together with parameters *F*_0_, *F*_k_, *M*_0_, *κ*_p_, *κ*_k_, *κ*_b_, *θ*_0_, *x*_j_, *x*_k_, *L*/2 and initial conditions (5a)–(5f) provide a unique solution and thus a unique shape of the bridging fibre and the k-fibres.

### Inputs and outputs of the model

In our study, we were not able to measure four parameters: *F*_0_, *F*_k_, *M*_0_, *x*_j_. Because parameters *F*_0_, *F*_k_, *x*_j_ are biologically relevant, we introduce geometrical constraints as an additional input to the model, whereas these three parameters were considered as an output of the model. First, we impose reflection symmetry on the system around its midpoint,


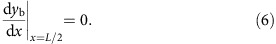


Second, the height of the end point of the second rod, *h*_k_, is constrained by the experimentally measured values. Third, we measured the orientation of the MT fibre in the vicinity of the kinetochore, *θ*_k_, which constraints the slope of the second rod at its end point,


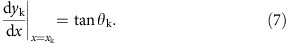


### Calculations for theoretical figures

To obtain Fig. 3b and Supplementary Fig. 3, we numerically solved equations (2)–(4), 4, 5 with conditions given by equations (5a–f) for variable parameters *F*_0_, *F*_k_, *M*_0_, *x*_j_, with the parameters *κ*_p_, *κ*_k_, *κ*_b_, *θ*_0_, *x*_k_, 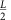 fixed by the experiment, until the values of *h*_k_, *θ*_k_ are consistent with the experiment, and the condition (6) fulfilled. For obtaining Fig. 4a, we numerically solved equations (2)–(4), 4, 5 with conditions given by equations (5a–f) for variable values of parameter *F*_0_, until the condition (6) is fulfilled, whereas the other parameters *F*_k_, *M*_0_, *κ*_p_, *κ*_k_, *κ*_b_, *θ*_0_, *x*_j_, *x*_k_, 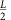 are fixed. Note that these complications are because of the implicit definition of the model. Flexural rigidities obey *κ*_p_=*κ*_k_+*κ*_b_ as explained in the section ‘Flexural rigidities of the rods'.

### Numerical methods

Equations (2)–(4), 4, 5 were solved numerically by the midpoint method. Solutions were obtained by using a step size Δ*x*=(*x*_f_−*x*_i_)/50, where *x*_i_ and *x*_f_ are the end points of the interval for the respective equation. Computer code is available on request.

### Cell culture and sample preparation for HeLa cells

HeLa-TDS cells were permanently transfected and stabilized (courtesy of Mariola Chacon) using pEGFP-α-tubulin plasmid, which was acquired from Frank Bradke (Max Planck Institute of Neurobiology, Martinsried). HeLa-Kyoto BAC lines stably expressing PRC1-GFP or Kif11-GFP were courtesy of Ina Poser and Tony Hyman (MPI-CBG, Dresden). Cells were grown in DMEM (1 g l^−1^ D-glucose, L-glutamine, pyruvate) with 50 μg ml^−1^ geneticin (Life Technologies, Waltham, MA, USA) and appropriate supplements. The cells were kept at 37 °C and 5% CO_2_ in a Heracell humidified incubator (Thermo Fisher Scientific, Waltham, MA, USA).

HeLa cells were transfected by electroporation using Nucleofector Kit R with the Nucleofector 2b Device, using the high-viability O-005 programme (Lonza, Basel, Switzerland). Transfection protocol provided by the manufacturer was followed. HeLa cells were transfected with mRFP-CENP-B plasmid (pMX234) provided by Linda Wordeman (University of Washington). In all, 1 × 10^6^ cells and 2 μg of plasmid DNA were used. Transfection was performed 24–36 h before imaging. Transfection of PRC1-GFP BAC line cells with mCherry-tubulin (1.5 μg of DNA) and mRFP-CENP-B (1.5 μg DNA) was performed 48 h before imaging.

For PRC1 siRNA, 1 × 10^6^ cells at ∼50–60% confluency were transfected with 200 nM raw targeting or non-targeting siRNA constructs diluted in a Nucleofector solution together with 1.5 μg mRFP-CENP-B plasmid. Transfection was performed by electroporation using Nucleofector Kit R with the Nucleofector 2b Device, using the high-viability O-005 programme (Lonza). The constructs used were as follows: siGENOME SMART pool for human PRC1 (M-019491-00-0005) and siGENOME control pool (D-001206-13-05), both from Dharmacon (Lafayette, CO, USA). Forty-eight hours after transfection, synchronization of transfected cells was started, as described below.

To prepare samples for microscopy, following the transfection, HeLa cells were seeded and cultured in 1.5 ml DMEM medium with supplements (without geneticin) at 37 °C and 5% CO_2_ on non-coated 35-mm glass coverslip dishes (MatTek Corporation, Ashland, MA, USA). Before live-cell imaging, the medium was replaced with Leibovitz's L-15 CO_2_-independent medium supplemented with fetal bovine serum (Life Technologies). For the experiments with fixed cells, cells were fixed in ice-cold methanol for 3 min and then washed three times with PBS and imaged immediately after fixation.

### Cell culture and sample preparation for PtK1 cells

PtK1 cells were cultured in HAM's F-12 medium (Invitrogen) supplemented with 5% sodium pyruvate (Invitrogen), 1% antibiotic–antimycotic (Invitrogen) and 10% fetal bovine serum (Invitrogen), and maintained at 37 °C in a humidified CO_2_ incubator. For experiments, cells were seeded in 35-mm glass coverslip dishes (MatTek Corporation) 24 or 48 h before observation. The cells were treated with 3 μM nocodazole (Sigma-Aldrich, St Louis, MO, USA) for 60–90 min. To visualize MTs, X-Rhodamine-labelled tubulin at a final concentration of 0.5 μg μl^−1^ in the injection buffer (20 mM HEPES, 100 mM KCl and 1 mM DL-dithiothreitol in H_2_O) was injected by using the semiautomatic mode of a microinjector (InjectMan NI 2, Eppendorf, Hamburg, Germany). To prevent tubulin polymerization in the needle, microinjection was performed at room temperature. Nocodazole was washed out and the cells were incubated at 37 °C for ∼15 min before imaging.

### Imaging and laser ablation

HeLa cells were imaged by using a Zeiss LSM 710 NLO inverted laser scanning microscope with a Zeiss PlanApo × 63/1.4 oil immersion objective (Zeiss, Jena, Germany) heated with an objective heater system (Bioptechs, Butler, PA, USA). During imaging, cells were maintained at 37 °C in Tempcontrol 37-2 digital Bachhoffer chamber (Zeiss). For excitation, a 488-nm line of a multiline Argon-Ion laser (0.45 mW; LASOS, Jena, Germany) and helium-neon (HeNe) 594 nm laser (0.11 mW) were used for GFP and RFP/mCherry, respectively. Spectral array detector from 34-Channel QUASAR Detection Unit (Zeiss) was used for detection of fluorescent light. Emission wavelengths for simultaneous image acquisition were selected by the sliding prisms incorporated in the detection unit. GFP and RFP/mCherry emissions were detected in ranges of 490–561 and 597–695 nm respectively. No images were acquired during laser ablation. According to Nyquist theory *xy* pixel size was set to 81 nm. Pinhole diameter was set to 0.7 μm (1 arbitrary unit). Pixel dwell time was 1 μs. Z-stacks were acquired at six focal planes with 0.5-μm spacing. The thickness of the optical sections was 700 nm. Image acquisition was performed for 20–40 time frames with 3.5–4.5 s intervals using unidirectional scanning. A titanium-sapphire (Ti:Sa) femtosecond pulsed laser (Chameleon Vision II, Coherent, Santa Clara, CA, USA) was utilized at a wavelength of 800 nm for MT severing (for discussion on laser sources for MT severing see ref. 41). The beam was coupled to the bleaching port of the microscope. The pulsed laser light was reflected on the objective with a long pass dichroic mirror LP690. Ablation was performed on user-defined, ellipse-shaped region of interest, ∼0.3 μm wide and 1 μm long with the major axis perpendicular to the k-fibre. The system was controlled with the ZEN 2010 software (Zeiss). Successful k-fibre severing was identified by observing at least one of the following events: decrease in the distance between sister kinetochores; reorientation of the severed k-fibre stub; and later (∼15 s after ablation) directed movement of the k-fibre stub tip towards the pole accompanied by increase in the distance between sister kinetochores.

PtK1 cells were imaged by using an Olympus IX71 inverted microscope with a Yokogawa CSU10 spinning-disc scan head (Yokogawa Electric Corporation, Tokyo, Japan), equipped with a fast piezo objective z-positioner (PIFOC, Physik Instrumente GmbH & K.G., Karlsruhe, Germany) and an Olympus UPlanSApo × 100/1.4 numerical aperture (NA) oil objective (Olympus, Tokyo, Japan). GFP and X-Rhodamine fluorescence was excited at 488 and 561 nm, respectively. Images were taken at a 2–3-s time interval. The laser intensity was controlled using the acousto-optic tunable filter inside the Andor Revolution Laser Combiner (ALC, Andor Technology, Belfast, UK). The emission wavelength was selected using respective emission filters BL 525/30 (Semrock, Rochester, NY, USA) and ET 605/70 (Chroma, Bellows Falls, VT, USA) mounted in a fast, motorized filter wheel (Lambda-10B, Sutter Instrument Company, Novato, CA, USA). The images have a *xy* pixel size of 168 nm. MT severing was performed using a MicroPoint (ALC, Andor Technology) with a 408-nm dye resonator cell.

### Synchronization of HeLa cells

Cell were seeded at ∼40% confluency in a poly-d-lysine-coated 35-mm glass coverslip dishes, No 1.5 coverglass (0.16–0.19 mm; MatTek Corporation) with 2 ml DMEM medium with supplements. At 4 pm the day before imaging, thymidine (Sigma-Aldrich) was added at a final concentration of 2 mM. Cells were left in thymidine for 17 h, and at 9 am each dish was washed three times with warm PBS and 2 ml of fresh DMEM medium with supplements was added. At 12:30 pm, RO-3306 (Calbiochem, Merck Millipore, Billerica, MA, USA) was added at a final concentration of 9 μM. At 7 pm, the dishes were washed three times with warm PBS. Then, the cells were left in the incubator with 2 ml DMEM medium with supplements for 30 min to recover. At 7:30 pm, the medium was replaced with L-15 with appropriate supplements and 20 μM of the proteasome inhibitor MG-132 (Sigma-Aldrich) to arrest the cells in metaphase. Imaging was started 15–20 min after adding MG-132.

### Immunostaining and imaging of immunostained PtK1 cells

For kinetochore and tubulin immunostaining, PtK1 cells were briefly rinsed in PBS with 5 mM EGTA and then fixed in ice-cold 95% methanol with 5 mM EGTA for 5 min at room temperature and, subsequently, for 30 min at −20 °C. Cells were then washed four times in PBS. Next, a 1-h block in 10% boiled goat serum at room temperature was followed by overnight incubation at 4 °C with primary antibodies diluted in 5% boiled goat serum in PHEM buffer. Cells were then washed four times in PBS with 0.1% Tween 20 (PBST), incubated with secondary antibodies for 45 min, washed in PBST and mounted on microscope slides with an anti-fading solution containing 90% glycerol, 10% Tris buffer and 0.5–1% *n*-propyl gallate. Primary antibodies were diluted as follows: human ACA (cat. 15–234, Antibodies Inc.), 1:100 and mouse anti-tubulin (cat. T9026, clone DM1A, Sigma-Aldrich), 1:500. Secondary antibodies were diluted as follows: Red-X-goat anti-human (cat. 109-295-088, Jackson ImmunoResearch Laboratories Inc.), 1:100 and Alexa Fluor 488 goat anti-mouse (cat. A11029, Invitrogen), 1:400.

Immunostained PtK1 cells were imaged on a swept field confocal unit (Prairie Technologies) attached to a Nikon Eclipse TE-2000U inverted microscope. The microscope was equipped with a × 100/1.4 NA Plan-Apochromatic phase-contrast objective lens, phase-contrast transillumination, transmitted light shutter and automated ProScan stage (Prior Scientific). The confocal head was accessorized with multiband pass filter set for illumination at 405, 488, 561 and 640 nm, and illumination was obtained through an Agilent monolithic laser combiner (MLC400) controlled with a four-channel acousto-optic tunable filter. Digital images were acquired with a HQ2 charge-coupled device camera (Photometrics). Acquisition time, *z* axis position, laser line power and confocal system were all controlled using the NIS Elements AR software (Nikon Instruments Inc.) on a PC computer (Dell). Z-series optical sections were obtained at 0.6-μm steps.

### Immunostaining of HeLa cells

Cells were fixed in ice-cold methanol (100%) for 3 min and washed. To permeabilize cell membranes, cells were incubated in triton (0.5% in PBS) for 25 min at room temperature. Unspecific binding of antibodies was blocked in blocking solution (1% normal goat serum (NGS) in PBS) for 1 h at 10 °C. Cells were incubated in 250 μl of the primary antibody solution (4 μg ml^−1^ in 1% NGS in PBS) for 48 h at 10 °C. Rabbit polyclonal anti-PRC1 antibody (H-70; sc-8356, Santa Cruz Biotechnology, USA) was used. After washing of the primary antibody solution, cells were incubated in 250 μl of the secondary antibody solution (4 μg ml^−1^ in 2% NGS in PBS; Alexa fluor 555 F (ab′) 2 fragment of goat anti-rabbit IgG (H+L), A21430, Molecular Probes, USA) for 1 h at room temperature, protected from light. After washing of the secondary antibodies, cells were incubated with 4,6-diamidino-2-phenylindole (1 μg ml^−1^ in PBS) for 5 min at room temperature and washed. After each incubation step, washing was performed three times for 5 min in PBS softly shaken at room temperature.

### Generation of 2xGFP-EB3/mCherry-CENP-A U2OS cells

EB3 and CENP-A were inserted with XhoI/EcoRI into pBabe GFP blastocidin-resistant and pBabe mCherry puromycin-resistant, respectively. A second GFP was inserted into a pBabe blastocidin GFP-EB3 vector upstream of GFP using BspEI/XhoI. The plasmids were transfected with a vesicular stomatitis virus envelope G protein (VSVG) viral coat plasmid in GP293 cells using Effectene according to the manufacturer's instructions (Qiagen). After 3 days, the supernatant containing viral particles was collected and added to U2OS cells. Cells expressing stably 2 × GFP- EB3 and mCherry-CENP-A were then selected using blastocidin and puromycin.

### Image analysis

Image processing was performed in ImageJ (National Institutes of Health, Bethesda, MD, USA). Quantification and statistical analysis were performed in MatLab (MathWorks, Natick, USA).

Kinetochores were tracked using Low Light Tracking Tool, an ImageJ plugin^42^. Tracking of kinetochores in the *xy* plane was performed on individual imaging planes or on maximum-intensity projections of up to three planes. Position in *z* direction was ignored because it had small contribution to the kinetochore movement. mRFP signal from kinetochores and mCherry signal from MTs in cells expressing PRC1-GFP, mCherry-tubulin and mRFP-CENP-B were acquired in the same channel and therefore kinetochores were tracked manually in these cells using the ImageJ manual tracking tool.

The signal intensity of a cross-section of a bridging fibre was measured in ImageJ by drawing a 3-pixel-thick line between outermost sister kinetochores and perpendicular to the line joining the centres of the two kinetochores. The measurements were made on the last image before cutting on the bridging fibre associated with the k-fibre that was cut. The intensity profile was taken along this line and the mean value of the background signal present in the cytoplasm was subtracted from it. The signal intensity of the bridging fibre was calculated as the area under the peak closest to the kinetochores. The width of this peak at the bottom of the peak was typically 0.6 μm. The signal intensity of the k-fibre was measured in a similar manner, 1 μm away from a kinetochore and perpendicular to and crossing the corresponding k-fibre. The width of the peak of the k-fibre at the bottom of the peak was typically 1 μm. In cells expressing PRC1-GFP, mCherry-tubulin and mRFP-CENP-B, measurements were made on four cells where laser-cutting was performed and subsequently the k-fibres and the bridging fibre separated, which allowed for separation of the kinetochore signal from that of the bridging fibre. The mCherry-tubulin signal of the bridging fibre overlapped with the PRC1-GFP signal, and we defined the width of the former by the width of the latter signal. In addition to these four cells, 11 measurements were made on fixed cells in one imaging plane or on a maximum-intensity projection of up to two planes by the procedure described above for other cell lines. Because kinetochores were not labelled in these cells, we measured the bridging fibre intensity in the mCherry channel on the position that corresponds to the midpoint of the same structure in the PRC1-GFP channel. By following a similar rationale, the distance between the kinetochore and the bridging fibre, *d*_bk_, was measured as the distance between the peaks of the curves representing the intensity profiles of the bridging fibre and of the kinetochores (Supplementary Fig. 1h).

The angles between the k-fibre and the long axis of the spindle in the vicinity of the spindle pole, *θ*_0_, and in the vicinity of the kinetochore, *θ*_k_, were calculated by fitting a line through three points on the measured contour of the k-fibre. A typical distance between the neighbouring points on the contours was ∼250 nm. Stub length was measured in the first frame after the cut from the centre of the corresponding kinetochore to the end of the ablated stub.

Graphs were generated in Matlab. ImageJ was used to scale images and adjust brightness and contrast. Figures were assembled in Adobe Illustrator CS5 and Adobe Photoshop CS5 (Adobe Systems, Mountain View, CA, USA). Data are given as mean±s.e.m., unless otherwise stated.

## Additional information

**How to cite this article:** Kajtez, J. *et al.* Overlap microtubules link sister k-fibres and balance the forces on bi-oriented kinetochores. *Nat. Commun.* 7:10298 doi: 10.1038/ncomms10298 (2016).

## Supplementary Material

Supplementary Information Supplementary Figures 1-4

Supplementary Movie 1 Laser-cutting of a k-fiber in a HeLa cell expressing GFP-tubulin (green) and mRFP-CENP-B (magenta). After the cut, which was done at time 0, the bridging fiber moved together with sister kinetochores, the intact k-fiber, and the cut k-fiber stub away from the spindle center. Scale bar represents 1 μm. The movie corresponds to still images from Fig. 1b.

Supplementary Movie 2 Laser-cutting of a k-fiber in a PtK1 cell expressing Hec1-GFP (shown in magenta), which was injected with X-Rhodamine-tubulin (shown in green). After the cut, which was done at time 0, the bridging fiber moved together with sister kinetochores, the intact k-fiber, and the cut k-fiber stub away from the spindle center. Scale bar represents 1 μm. The movie corresponds to still images from Fig. 1j.

Supplementary Movie 3 Laser-cutting of a k-fiber in a HeLa cell expressing PRC1-GFP (green) and mRFP-CENP-B (magenta). After the cut, which was done at time 0, the PRC1 signal in the bridging fiber moved together with sister kinetochores away from the spindle center. Scale bar represents 1 μm. The movie corresponds to still images from Fig. 2a.

Supplementary Movie 4 Laser-cutting of a k-fiber in a U2OS cell expressing 2xGFP-EB3 (green), mCherry-CENP-A (magenta) and mCherry-tubulin (magenta). Numerous EB3 comets (green spots) can be seen. Note that occasionally comets can be seen passing between the outermost sister kinetochores. Scale bar represents 1 μm. The movie corresponds to still images from Fig. 2d,e.

Supplementary Movie 5 Laser-cutting of a k-fiber in a HeLa cell expressing PRC1-GFP (green), mRFP-CENP-B (magenta) and mCherry-tubulin (magenta). After the cut, which was done at time 0, the bridging fiber moved together with sister kinetochores, the intact k-fiber, and the cut k-fiber stub away from the spindle center. Note that this movement is faster than in Supplementary Video 1. Scale bar represents 1 μm. The movie corresponds to still images from Fig. 4c.

## Figures and Tables

**Table 1:** Experimentally measured parameters of bridging MTs and spindle shape.

**Parameter**	**HeLa cells**	**PtK1 cells**
Number of MTs in the bridging fibre, *n*_b_	14±2 (*n*=37)	6±1 (*n*=30)
Angle between the k-fibre and the long axis of the spindle in the vicinity of the spindle pole, *θ*_0_ (°)	65.5±8.8 (*n*=23)	52.6±8.4 (*n*=30)
Angle between the k-fibre and the long axis of the spindle in the vicinity of the kinetochore, *θ*_k_ (°)	13.7±10.1 (*n*=23)	21.2±10.2 (*n*=30)
Distance between the kinetochore and the bridging fibre, *d*_bk_ (μm)	0.24±0.15 (*n*=42)	0.20±0.10 (*n*=23)
Distance between sister kinetochores, *d*_k_ (μm)	1.05±0.32 (*n*=52)	1.92±0.54 (*n*=50)
Spindle length, *L* (μm)	11.1±1.2 (*n*=52)	11.8±1.7 (*n*=50)
Spindle half-width*, h*_k_ (μm)	5.0±0.7 (*n*=52)	4.0±0.6 (*n*=50)

MT, microtubule.

All values are given as mean±s.d., with the number of measurements in brackets. *θ*_0_ and *θ*_k_ are the angles between the k-fibre and the long axis of the spindle, calculated 1 μm away from the spindle pole and at the end of the k-fibre (that is, at the kinetochore), respectively. The distance *d*_bk_ is positive if the kinetochore is on the outer side of the bridging fibre, with respect to the spindle centre. Spindle length *L* was calculated as the distance between the spindle poles. Spindle half-width *h*_k_ was calculated from the midpoint between the outermost sister kinetochores to the midpoint between the spindle poles.
