# Supplementary material for: Overlap microtubules link sister k-fibres and balance the forces on bi-oriented kinetochores
Source: Nat Commun. 2016 Jan 5;7:10298. doi: 10.1038/ncomms10298 (PMC4728446; doi:10.1038/ncomms10298)
Supplement: Supplementary Information — Supplementary Figures 1-4 [file ncomms10298-s1.pdf]

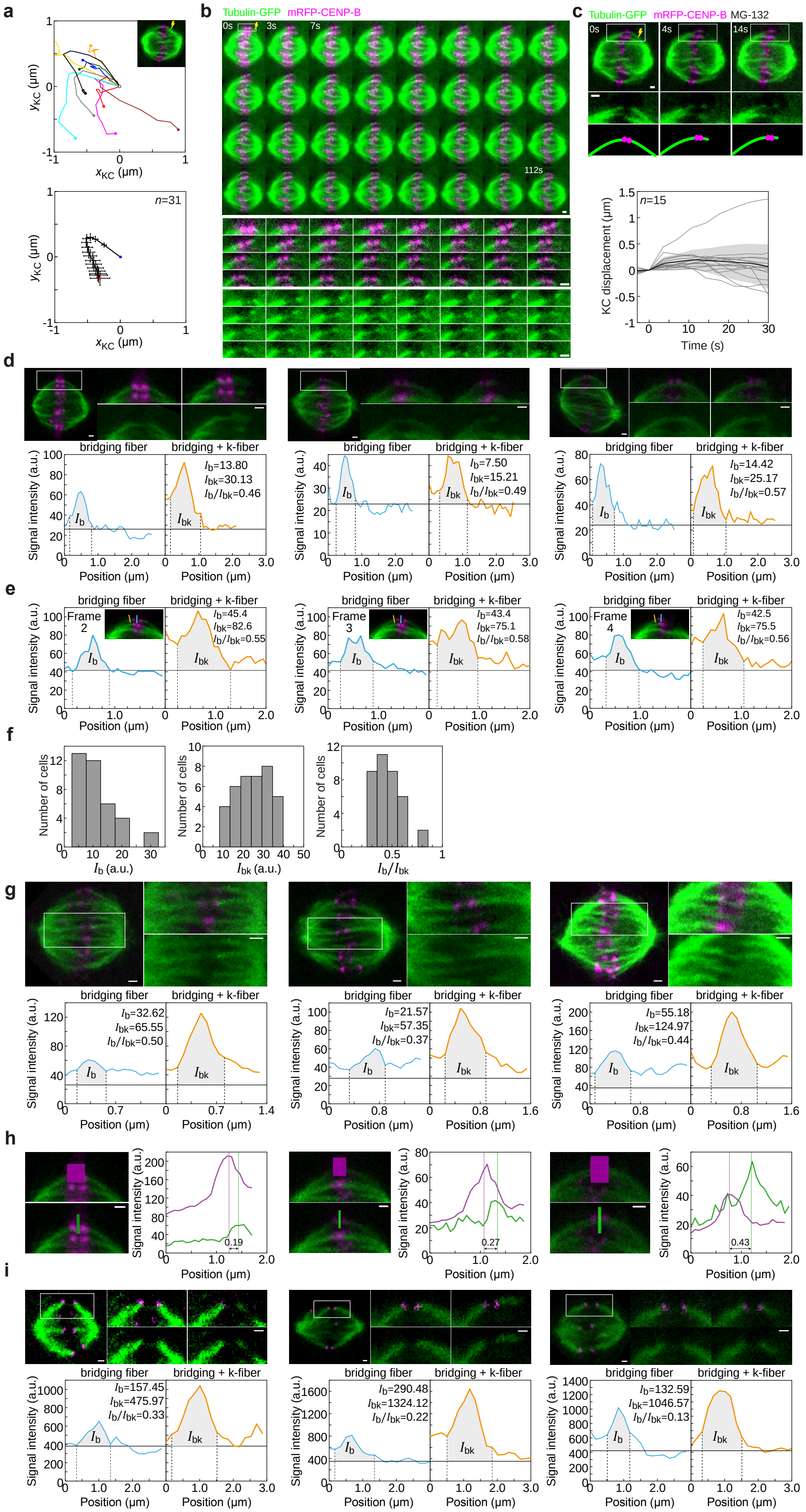

a
b
Tubulin-GFP mRFP-CENP-B
c
Tubulin-GFP mRFP-CENP-B MG-132
d
e
f
g
h
i
n=31
n=15
KC displacement (μm)
Time (s)
bridging fiber
bridging + k-fiber
Signal intensity (a.u.)
Position (μm)
Number of cells

Supplementary Figure 1 Additional characterization of bridging MTs.

(**a**) Two-dimensional trajectory of the kinetochore that was closer to the cutting site, during the first minute after the cutting, in HeLa cells expressing tubulin-GFP and mRFP-CENP-B. Upper panel: 10 examples of trajectories, lower panel: mean trajectory of kinetochores from *n*=31 cells, error bars represent s.e.m. The starting point of all trajectories has been set to (0,0). The coordinate system is oriented in the same manner as the spindle image in the inset (yellow sign marks the cut).

(**b**) Time-lapse images of a metaphase HeLa cell expressing tubulin-GFP (green) and mRFP-CENP-B (magenta), covering 112 seconds. The k-fiber was cut after the first image at the location marked by the yellow lightning sign. Top: images of the whole spindle; middle and bottom: images of the region inside the white box, showing both channels and green channel only, respectively. Note that the bridging fiber, the intact k-fiber, and the k-fiber stub extending from the kinetochore closer to the cutting site moved together with sister kinetochores, first in the direction outwards from the spindle and later back towards the spindle. The observation that these structures move together both outwards and inwards shows that the bridging fiber, sister k-fibers and sister kinetochores are mechanically coupled into a single object.

(**c**) Time-lapse images of the spindle (upper row) in a HeLa cell expressing tubulin-GFP (green) and mRFP-CENP-B (magenta), which was treated with MG-132 (see Methods). Enlargements of the region inside the white rectangle in the green channel (middle row) and the corresponding drawings (bottom row) are shown. The outermost k-fiber was cut with a laser (yellow lightning sign) at time 0. The graph at the bottom shows the displacement of the kinetochore that was closer to the cutting site in the direction perpendicular to the long axis of the spindle, with respect to its position before the cutting. Individual cells, the mean value and the standard deviation are shown by thin lines, thick black line and the shaded region, respectively; *n* denotes the number of cells.

(**d**) Three examples (from left to right) of the measurement of the bridging fiber signal intensity in HeLa cells expressing tubulin-GFP (green) and mRFP-CENP-B (magenta). In each example, the image at the left shows the spindle, while the four images to the right of the spindle show enlargements of the boxed area (top: both channels, bottom: green channel only; left: before cutting, right: after cutting). The graphs show tubulin-GFP signal intensity of the bridging fiber, I_b (blue), and the bundle consisting of the bridging fiber and the k-fiber, I_{bk} (orange), which were measured as described in Methods (see also Fig. 1d). Horizontal lines mark the background signal, vertical lines delimit the area (grey) where the signal was measured. The values of the measured signal intensities are given in the graphs.

(**e**) Three examples of measurements of the tubulin-GFP signal intensity of the bridging fiber, I_b (blue, measured along the blue line in the image) and the bundle consisting of the bridging fiber and the k-fiber, I_{bk} (orange, measured along the orange line in the image) in a HeLa cell expressing tubulin-GFP (green) and mRFP-CENP-B (magenta). The measurements were performed on frames 2, 3, and 4 of the movie of a single cell, as denoted in the graphs. Horizontal lines mark the background signal, vertical lines delimit the area (grey) where the signal was measured. The values of the measured signal intensities, which are given in the graphs, show that the variation in the measured signals in the consecutive images is roughly 10%.

(**f**) From left to right: distribution of the measured signal intensity of the bridging fiber, I_b, the signal of the bundle consisting of the bridging fiber and the k-fiber, I_{bk}, and the ratio of these intensities, I_b/I_{bk}. Note that the distribution of I_b/I_{bk} is narrower than the distributions of I_b and I_{bk}.

(**g**) Three examples of the bridging fiber signal intensity in the interior of the spindle. The bridging fibers in the interior of the spindle were defined as those for which the distance between

the center of the bridging fiber and the long axis of the spindle was smaller than 3 µm. In total, 27 bridging fibers from 20 cells were measured and the resulting I_b/I_{bk} was 46±1%. Legend as in panel **d**, except for the fact that the k-fibers were not cut.

(**h**) Measurement of the distance between the kinetochore and the bridging fiber, d_{bk}, in the cells shown in panel **d**. The signal intensity of the kinetochores (purple curve) was measured along the purple line in the image, which was typically 17 pixels wide and started outside of the spindle (position 0), by using the maximum intensity over the width of the line. The signal of the bridging fiber (green curve) was measured along the 3 pixel wide line (green line in the image), which was positioned at the center of the purple line. The distance between the kinetochore and the bridging fiber was measured as the distance between the peaks (vertical lines) of the two curves. The resulting values are given in the graphs.

(**i**) Three examples of the measurement of the bridging fiber signal intensity in PtK1 cells expressing Hec1-GFP (magenta), injected with X-Rhodamine-tubulin (green). Legend as in panel **d**.

Scale bars in all images represent 1 µm.

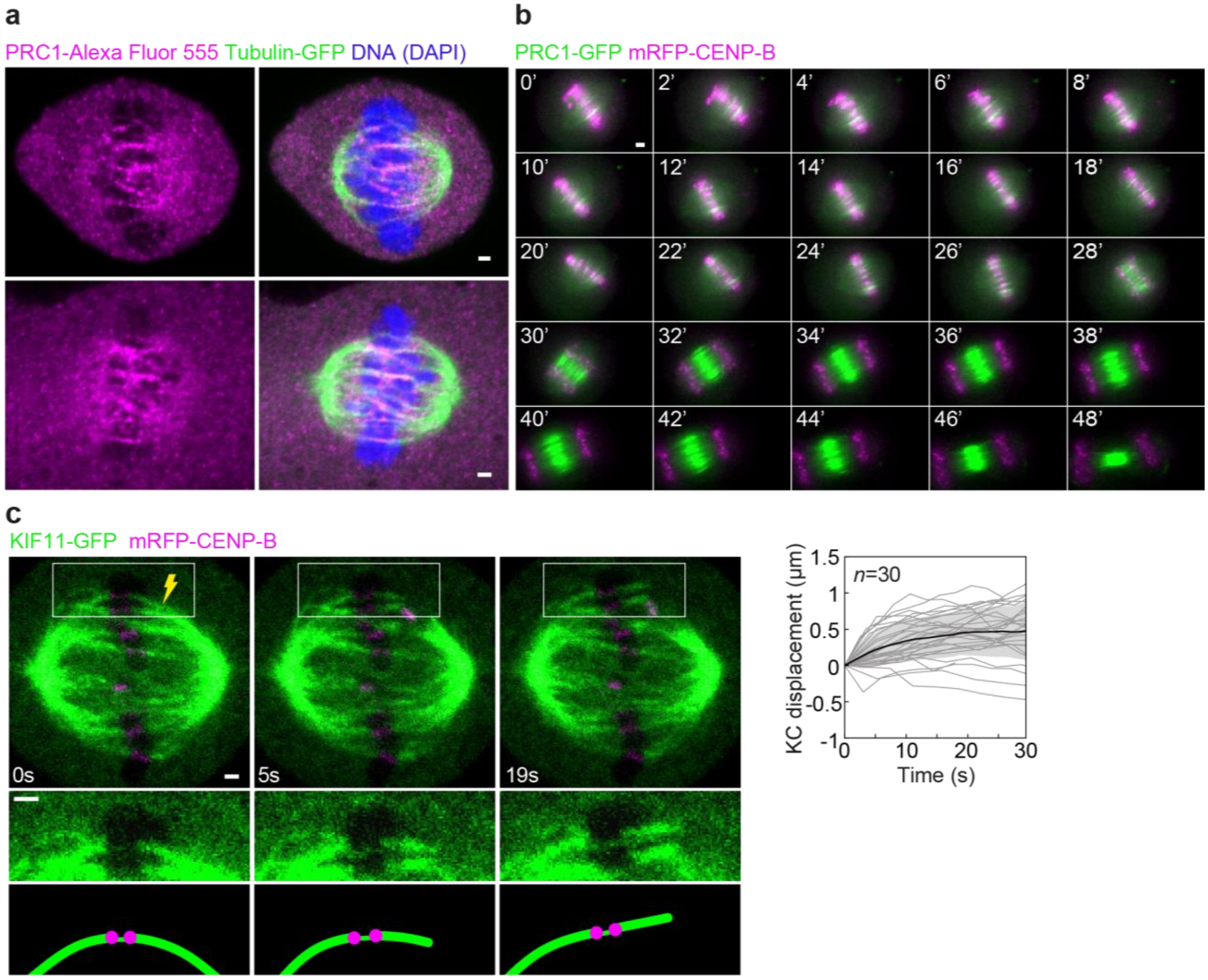

Supplementary Figure 2 Localization of PRC1 in metaphase and anaphase, and laser-cutting in cells expressing KIF11-GFP.

(**a**) Immunostaining of PRC1 shows that endogenous PRC1 (magenta) is present along the MTs in the central part of the spindle at metaphase in HeLa cells stably expressing tubulin-GFP (green). Images at the left show PRC1 signal only (magenta), whereas images at the right show PRC1 (magenta), tubulin (green) and chromosomes labeled with DAPI (blue) in the same cell. The immunostaining protocol is described in Methods.

(**b**) Dynamics of PRC1 in metaphase and anaphase in a cell stably expressing PRC1-GFP (green) from a BAC and mRFP-CENP-B (magenta). PRC1-GFP signal is visible in the central part of the spindle during metaphase (0-26 min), marking the overlap zones of anti-parallel MTs. The signal increases significantly in anaphase (from 30 min onwards).

(**c**) Left: Images of the spindle (upper row) in a HeLa cell expressing KIF11-GFP (green) and mRFP-CENP-B (magenta). Enlargements of the region in the white rectangle (middle row: KIF11-GFP, bottom: scheme). The outermost k-fiber was cut with a laser (yellow lightning sign) at time 0. Right: Displacement of the kinetochore that was closer to the cut site in the direction perpendicular to the long axis of the spindle, with respect to its position before the cutting. Individual cells, the mean value and the standard deviation are shown by thin lines, thick black line and the shaded region, respectively; *n* denotes the number of cells.

All scale bars are 1 μm.

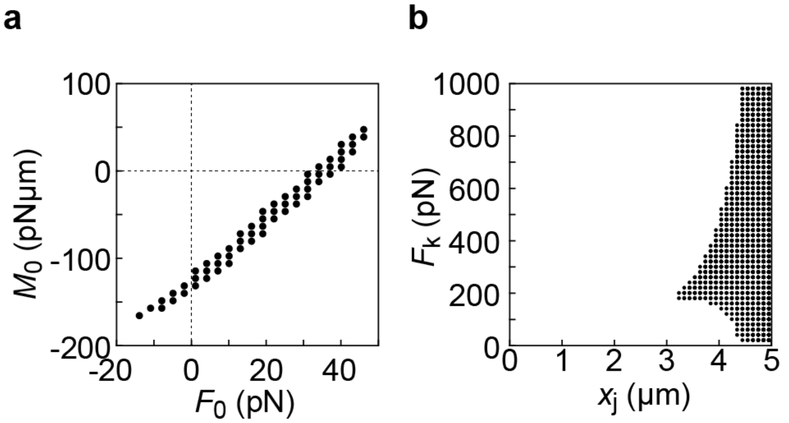

Supplementary Figure 3 Predictions of the model for the bending moment and force at the pole (**a**), and the force at the kinetochore and the junction (**b**).

(**a**) and (**b**) In the parameter space, we plot only those parameter values for which the calculated spindle shape reproduces the measured spindle shape within experimental variability (Table 1). In particular, the angle at the pole is constrained within interval $\theta_0 = 56.7° - 74.3°$, the angle at the kinetochore is constrained within interval $\theta_\mathrm{k} = 3.6° - 23.8°$, the vertical position of the kinetochore is constrained within interval $h_\mathrm{k} = 4.3$ μm $- 5.7$ μm. The length of the spindle and the horizontal position of the kinetochore are fixed to $L = 11.1$ μm and $x_\mathrm{k} = 5.05$ μm, respectively. The number of MTs in the bridging fiber is $n_\mathrm{b} = 14$ and the number of MTs in the k-fiber is $n_\mathrm{k} = 17$. Flexural rigidity of a single MT is $\kappa_0 = 30$ pNμm^2. The unknown parameter, junction, is on the grid of 50 points, within physically allowed interval $x_\mathrm{j} = 0$ μm $- 5.05$ μm. In (**a**) the force at the pole and the bending moment at the pole are discretized with steps $\Delta F_0 = 3$ pN and $\Delta M_0 = 5$ pNμm, respectively. Force at the kinetochore F_k is discretized to four different values 80 pN, 160 pN, 240 pN and 320 pN. The parameters F_k and x_j are not shown in the plot and thus the plotted points may correspond to several values of these parameters. In (**b**) the force at the kinetochore is discretized with step $\Delta F_\mathrm{k} = 20$ pN. The force at the pole is on a grid of 10 points within the interval $F_0 = 30$ pN $- 40$ pN. Note that the points in the plot may correspond to several values of F_0. The bending moment at the pole M_0 is set to zero.

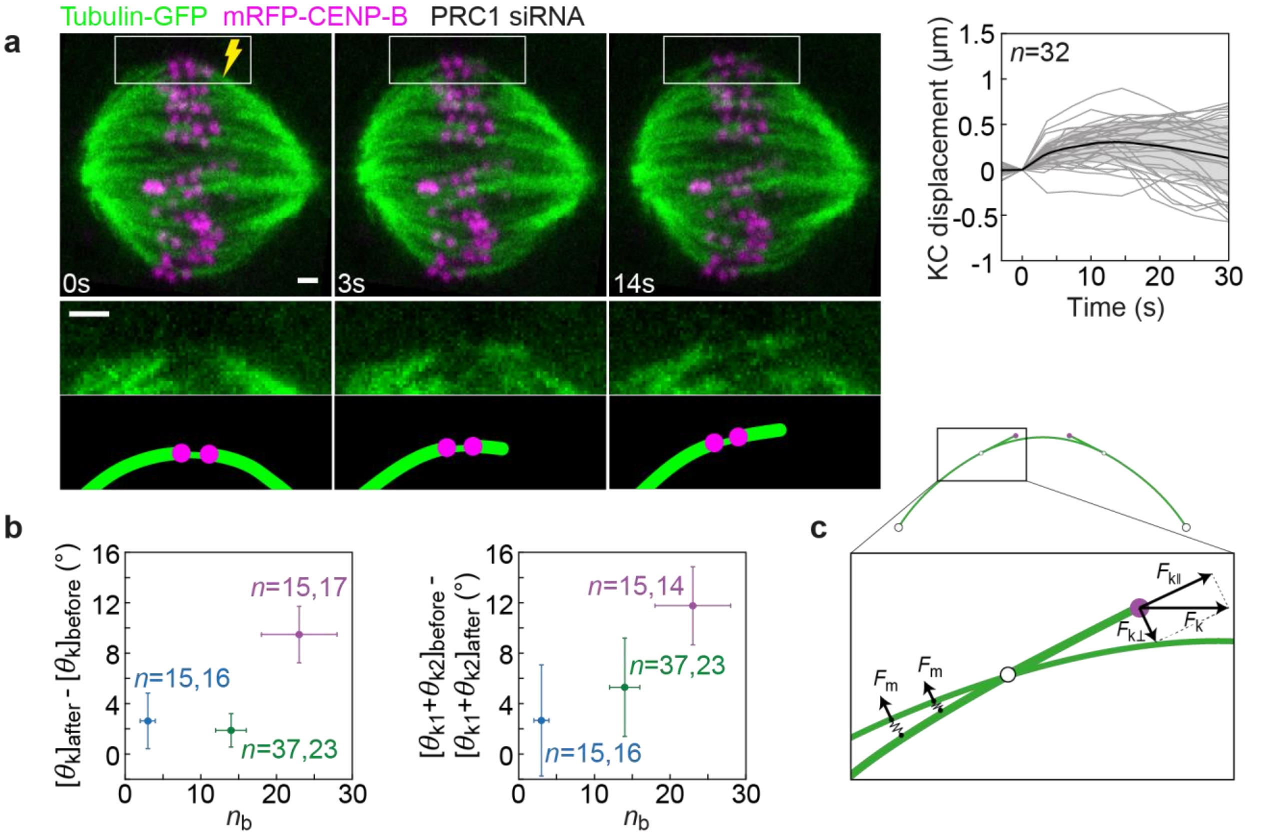

Supplementary Figure 4 HeLa cells treated with PRC1 siRNA, straightening of the k-fibers in cells with different numbers of bridging MTs, and the morphology of the junction.

(**a**) Analysis of HeLa cells treated with PRC1 siRNA. Left: Time-lapse images of the spindle (upper row) in a HeLa cell expressing tubulin-GFP (green) and mRFP-CENP-B (magenta), which was treated with PRC1 siRNA (see Methods). Enlargements of the region inside the white rectangle in the green channel (middle row) and the corresponding drawings (bottom row) are shown. The outermost k-fiber was cut with a laser (yellow lightning sign) at time 0. Scale bars, 1 µm. Right: Displacement of the kinetochore that was closer to the cut site in the direction perpendicular to the long axis of the spindle, with respect to its position before the cutting. Individual cells, the mean value and the standard deviation are shown by thin lines, thick black line and the shaded region, respectively; *n* denotes the number of cells. We measured a smaller inter-kinetochore distance, 0.52±0.04 µm (n=19), after treatment with PRC1 siRNA, compared with 0.82±0.05 µm (n=14) after treatment with non-targeting siRNA (p=0.00002).

(**b**) Straightening of the k-fibers in cells with different number of MTs in the bridging fiber. The difference in the angle between the intact k-fiber and the k-fiber stub (left) and the difference in the sum of θ_k of sister k-fibers (right) before and 4 seconds after the cutting is shown as a function of the number of MTs in the bridging fiber, n_b. Larger values on the ordinate mean a more pronounced straightening of the k-fibers. Three HeLa cell lines are shown: cells expressing PRC1-GFP, mRFP-CENP-B and tubulin-mCherry (magenta), cells expressing GFP-tubulin and mRFP-CENP-B (green), and cells expressing GFP-tubulin and mRFP-CENP-B, treated with PRC1 siRNA (blue). *n* denotes the number of cells, where the first and the second number

designate the number of cells of each cell line used to calculate the mean value on the abscissa and on the ordinate, respectively. Error bars represent s.e.m.

(**c**) Scheme of the region around the junction (box on the scheme above). Cross-linkers are drawn as small springs, the point where the bridging fiber crosses the k-fiber is marked by the white circle, and the kinetochore by the pink circle. Forces on the k-fiber are also depicted, see Discussion in the main text for details.
